# Glass Transition in Rice Pasta as Observed by Combined Neutron Scattering and Time-Domain NMR

**DOI:** 10.3390/polym13152426

**Published:** 2021-07-23

**Authors:** Magdalena Witek, Maciej Krzystyniak, Giovanni Romanelli, Teresa Witczak

**Affiliations:** 1 Department of Biotechnology and General Technology of Food, Faculty of Food Technology, University of Agriculture in Krakow, Balicka 122, 30-149 Krakow, Poland; magdalena.witek@urk.edu.pl; 2 ISIS Facility, Rutherford Appleton Laboratory, Chilton, Didcot OX11 0QX, UK; giovanni.romanelli@stfc.ac.uk; 3 Department of Engineering and Machinery for Food Industry, Faculty of Food Technology, University of Agriculture in Krakow, Balicka 122, 30-149 Krakow, Poland; teresa.witczak@urk.edu.pl

**Keywords:** glass transition, NMR relaxation, DSC, neutron diffraction, neutron transmission, neutron Compton scattering, average functional group approximation

## Abstract

Experimental protocols aiming at the characterisation of glass transition often suffer from ambiguity. The ambition of the present study is to describe the glass transition in a complex, micro heterogeneous system, the dry rice pasta, in a most unambiguous manner, minimising the influence of technique-specific bias. To this end, we apply an unprecedented combination of experimental techniques. Apart from the usually used NMR and DSC, we employ, in a concurrent manner, neutron transmission, diffraction, and Compton scattering. This enables us to investigate the glass transition over a range of spatio-temporal scales that stretches over seven orders of magnitude. The results obtained by neutron diffraction and DSC reveal that dry rice pasta is almost entirely amorphous. Moreover, the glass transition is evidenced by neutron transmission and diffraction data and manifested as a significant decrease of the average sample number density in the temperature range between 40 and 60 °C. At the microscopic level, our NMR, neutron transmission and Compton scattering results provide evidence of changes in the secondary structure of the starch within the dry rice pasta accompanying the glass transition, whereby the long-range order provided by the polymer structure within the starch present in the dry rice pasta is partially lost.

## 1. Introduction

The glass transition concept, adapted from the science of polymer materials, has established itself very well to test the quality and stability of concentrated foods, i.e., low moisture content foods. It has been very useful to understand the structure-function relationship in many complex polysaccharide-based materials and the effect of plasticization by water on their thermal and mechanical properties [1,2,3,4,5,6]. The main parameter pertaining to the glass transition is the glass transition temperature $T_{g}$. It is considered as a boundary in the phase diagram that separates non-equilibrium glassy state from a rubbery state [7]. Several physio-chemical and thermo-mechanical properties of food can change across a glass transition, and they form a physical basis of the determination of $T_{g}$ by techniques such as differential scanning calorimetry (DSC), thermomechanical analysis (TMA), dynamic mechanical analysis (DMA), rheological methods, or dielectric analysis. The evidence of structural differences between glasses and melts has also been revealed via X-ray scattering using radial distribution functions [8], atomic force microscopy [9], and the analysis of fifth-order susceptibility [10]. Other methods of glass transition detection, which allow for studying molecular dynamics, such as electron spin resonance (ESR) or nuclear magnetic resonance (NMR), have become very useful as well. Especially low-field NMR has proven to be a very potent tool for the characterization of the transition between the glass and rubbery state in many carbohydrate-based food materials. The method owes its high sensitivity to glass transition in food to dramatic changes in the spin-spin relaxation time ($T_{2}$), detected for the solid food component, or to changes in the spin-lattice relaxation times ($T_{1}$) [11,12,13]. The high sensitivity of the relaxation times to glass transition has allowed for the introduction of an entirely new concept in NMR studies of food, the NMR phase diagram, which has been extensively used in the context of shelf-life stability of complex food systems [14]. Interestingly, the concept of the NMR phase diagram is constantly evolving, with some recent studies suggesting the incorporation of the second moment $M_{2}$ of an NMR line as a phase-diagram variable, as evidenced by studies of the glass transition in amorphous sugars [15], amylose films [16] or starch systems [17]. Importantly, the temperature of change in the second moment and $T_{g}$ determined classically by the DSC method may differ by at least several degrees due to the fact that the glass transition temperature of a given system does not have a unique value but rather depends on the cooling rates used to observe $T_{g}$, sample composition and its processing [13,15,16,18]. What is more, the different analytical and spectroscopic methods have different spatial and temporal scales at which they detect the glass transition, thus leading to discrepancies in its temperature value. Therefore, the differences in $T_{g}$ values arise from the nature of the techniques used. To this end, two categories of techniques can be distinguished: those working in the linear regime, for instance, dynamic-mechanical analysis, and those where a non-linear perturbation is applied [7]. Additionally, in more complex food systems, a broader glass transition temperature range may be observed by different methods, or some methods may even fail to determine it.

In an attempt to shed more light on the phenomenon of glass transition in food and to overcome some of the methodological difficulties described above, in this work, we extend the usual arsenal of spectroscopic and analytical techniques used for its characterization and apply a suite of neutron spectroscopic and structural probes covering seven decades of temporal scales concurrently. We make such a broadband study of the phenomenon possible owing to the characteristics of the world-unique neutron instrument, VESUVIO thermal-to-epithermal neutron station, installed at the ISIS Neutron and Muon Source at the Rutherford Appleton Laboratory in the UK [19,20,21,22,23,24,25]. Traditionally, the core technique employed at VESUVIO has been neutron Compton scattering (NCS) [19,20,21,22,23]. Due to the recent upgrades in the instrumentation, sample environment, and software [19,20,24,26,27,28,29,30], VESUVIO has become a very versatile tool, enabling concurrent measurements using techniques such as NCS, energy-dependent neutron transmission [29,31,32,33,34,35,36,37,38,39], neutron diffraction [30,40,41], and gamma spectroscopy [24,42,43,44,45]. The aforementioned techniques, especially when applied concurrently, enable the investigation of a plethora of physical and chemical properties of condensed matter systems. As the full description of experimental protocols and their applicability to tackle specific problems related to structure-function relationships in materials is beyond the scope of this work, here, we will only concentrate on those aspects of VESUVIO-based techniques that are relevant for food science, especially in the context of the glass transition.

The NCS technique was employed to investigate the glassy dynamics already at the beginning of the VESUVIO user program more than two decades ago [46]. The choice of the technique for the investigation of nuclear dynamics of disordered, amorphous systems has been dictated by the fact that NCS measures momentum distributions (NMDs) of individual nuclei in a mass-resolved manner, no matter if the underlying vibrational densities of states (VDOS) stems from a completely ordered lattice or a disordered network of atoms [21,22]. As the integral intensity of each peak recorded in an NCS spectrum is proportional to the number of nuclei of a given type per formula unit, the method enables monitoring of the stoichiometry as a function of physical changes or chemical reactions [21,22]. In this spirit, the first study of glass by NCS led to the discovery of the softening of NMDs of individual nuclei induced by the local disorder [46], an observation that has been present in many other studies of disordered amorphous systems ever since and has become a universal spectroscopic fingerprint of the glassy dynamics [41,47].

Apart from NCS, the technique of incident neutron energy-dependent sample transmission has also proven to be very useful in the investigation of systems with not very well-defined long-range order, such as liquid solutions and polymers [26,34,35,48,49,50,51,52]. Neutron transmission is often chosen for the investigation of disordered systems due to its simplicity as an experimental technique and a lack of complicated numerical corrections that are necessary in the case of neutron spectroscopic techniques, including the NCS [26,34,35,48,49,50,51,52]. Neutron transmission on VESUVIO covers a vast range of incident neutron energies, from a fraction of a millielectronvolt up to thousands of electron volts. The initial part of the transmission curve, corresponding to the incident neutron energies in the range 1 meV to 1 eV, is shaped by a wide range of different types of excitations, from local to the long-range ones [29,36,49]. Moreover, owing to the capability of VESUVIO to detect the second part of the transmission curve, corresponding to the incident neutron energies above a few eV, the regime of the Impulse Approximation (IA) is reached, where each neutron transmission curve reaches a plateau with the magnitude of transmission determined solely by the product of the sample thickness, its number density, and the total free scattering cross-section weighted by the sample stoichiometry [21,22,29,31,34,35,36,39,49]. This last unique feature of VESUVIO enables measurements of changes of density in different regions of phase diagrams of condensed matter systems and molecules, particularly important in the context of the glass transition.

Finally, high-resolution neutron diffraction available on VESUVIO has enabled simultaneous monitoring of nuclear dynamics and structure across different thermodynamic conditions for a plethora of condensed-matter systems [21,22,24,30,31,40,41]. Importantly, in the context of disordered systems, owing to the relatively large incoherent neutron cross-section of a nucleus very abundant in organic matter, the hydrogen, neutron diffraction can be used indirectly to monitor the changes of the incoherent background present in diffraction curves that are dictated by the changes of the number density due to phase transitions.

Motivated by the recent developments described above and tapping on the unique potential of the VESUVIO neutron station, in this work, we employ neutron transmission, diffraction and Compton scattering concurrently to investigate structural and dynamic changes in a micro-heterogeneous, disordered system—the dry rice pasta—across a wide range of temperatures across the glass transition. Moreover, we correlate the temperature-driven changes in the magnitudes of neutron observables measured on VESUVIO with the apparent changes in the NMR and DSC observables in order to provide stronger evidence of a glass transition in this important food science and technology system.

## 2. Materials and Methods

### 2.1. Pasta Preparation and Composition

The pasta was prepared with rice flour (Balviten, Katowice, Poland) and water (37 g/100 g d.m.). The homogeneous pasta dough was obtained by kneading with a Thermomix (Verwork, Wuppertal, Germany) for 10 min. The dough was passed through a kitchen pasta machine (OWIM GmbH &Co. Kg, Neckarsulm, Germany) to get pasta in the shape of strips (6 × 2 × 100 mm). Fresh strips of pasta were then dried by air at 45 °C. Dried samples of pasta were fine-milled for all analyses.

The obtained samples of milled pasta of different moisture content were stored at 20 °C in equilibrium chambers at different levels of air relative humidity over a set of saturated solutions of the following salts: (humidity levels given in parentheses) LiCl (11.3%), CH_3_COOK (23.1%), MgCl_2_ (33.1%), K_2_CO_3_ (43.2%), Mg(NO_3_)_2_ (54.4%), CuCl_2_ (68.0%), NaCl (75.0%), KCl (85.1%), KNO_3_ (94.0%) and over P_4_O_10_. The moisture content (g H_2_O/g product) was determined by standard oven drying at 105 °C for 3 h, which for a dried sample of pasta was equal to 5.16 ± 0.03%. The protein content, measured by the Kjeldahl method using K-435 and B-324 combustion and distillation units (Büchi, Flawil, Switzerland) [53] was 7.02 ± 0.01%. The amount of fat was 0.45 ± 0.01% [54], whereas the total ash content was 0.73 ± 0.01% as obtained by combustion at 550 °C for 5 h. Total carbohydrates were estimated as a residue to value 86.64 ± 0.01%.

### 2.2. Differential Scanning Calorimetry

Glass transition temperatures of pasta were determined using a differential scanning calorimeter DSC 204F1 (Netzsch-Gerätebau, Selb, Germany). The instrument was calibrated using the following standards: Hg, In, Sn, Bi, Zn, and CsCl. Samples of different moisture content were loaded into an aluminium cell, and the cell was tightly sealed. Samples were analyzed at a DSC temperature program from −80 to 250 °C with a heating rate of 10 °C/min. A double scanning program was used for each sample in order to reduce the enthalpy relaxation of the amorphous samples. During the first heating scan, a target temperature is set to a value higher than that expected for the glass transition under consideration. During this scan, the sample material is thus allowed to relieve the stress developed during the storage of the sample in the glassy state. In the second scan, the physical state of the sample is already devoid of thermal history, and it is possible to detect the transformation associated only with the glass transition [55]. An empty aluminium pan of the same weight as the sample was used as a reference. The measurement was conducted at least three times for each sample. Glass transition thermal effects were determined on thermograms by using the Proteus Analysis software (Netzsch-Gerätebau, Selb, Germany). The initial point, mid-point, and end-point of glass transition temperatures were determined from heating thermograms. The mid-point transition temperature was assumed as the characteristic temperature of the glass transition.

The fitting of experimental data was performed using Statistica (StatSoft, Inc., Tulsa, OK, USA) software version 13.3. The parameters of the model were calculated by using non-linear regression with the Levenberg-Marquardt algorithm. The coefficient of determination (R2) was estimated to evaluate each model’s ability to describe experimental data.

### 2.3. NMR Measurements and Data Analysis

Samples of dry rice pasta with a moisture content of about 0.015 g/g d.m. were chosen for the NMR experiments. Proton relaxation measurements were performed using a Bruker Minispec NMR spectrometer (Bruker BioSpin, Poznan, Poland) operating at the resonance frequency of 60 MHz at stabilized temperatures in the range from 0 to 70 °C with a heating rate of 2.5 °C/min. The spin-spin relaxation decays were obtained by recording Free Induction Decay signals (FIDs). Each FID was sampled at 2048 points every 0.9 µs. The length of the 90° pulse was set to 2.0 μs. The repetition time between scans was 2 s.

Two models were tested to fit each recorded FID. The first model was inspired by the NMR studies, describing carbohydrate systems in a glassy state [16,17,18]:

(1) $$F_{1}(t)\;=\;S_{s}\exp\;\;\left(-{\left(\frac{t}{T_{2s}^{*}}\right)}^{2}\right)\frac{\sin(bt)}{bt}\;+\;L\exp\left(-\frac{t}{T_{2L}^{*}}\right).$$

The second model includes an additional Gaussian function accounting for the relaxation of another type of solid-like proton fraction in pasta, which can be expressed as:

(2) $$F_{2}(t)\;=\;S_{g}\exp\;\;\left(-{\left(\frac{t}{T_{2g}^{*}}\right)}^{2}\right)\;+\;S_{s}\exp\;\;\left(-{\left(\frac{t}{T_{2s}^{*}}\right)}^{2}\right)\frac{\sin(bt)}{bt}\;+\;L\exp\left(-\frac{t}{T_{2L}^{*}}\right).$$

The parameters associated with amplitudes and time constants accounting for the relaxation of the solid-like fraction of protons are ($S_{s}$, $T_{2s}^{*}$, b) and ($S_{g}$, $T_{2g}^{*}$, $S_{s}$, $T_{2s}^{*}$, b) for the first and second model, respectively. The liquid-like fraction of protons is described in both models by an exponential function, with L denoting its amplitude and $T_{2L}^{*}$ its spin-spin relaxation time.

In order to perform a critical appraisal of the models describing the spin-spin relaxation of protons in starch samples under investigation, a model-selection tool inspired by the Bayesian inference technique, FABADA [56,57,58], was employed within a bespoke Python script implemented within the MantidPlot computational environment [28,29,56,59]. FABADA analyses the distributions of residuals governed by the probability laws underlying the chi-square statistics and is much less likely to get stuck in local minima of the *χ*^2^ landscape as it usually happens with classical minimization procedures. Moreover, correlations between parameters are considered in a natural way. Finally, unlike in the traditional minimization methods, parameters are obtained as probability distribution functions. Additionally, this type of analysis yields the shapes of $\chi^{2}$ distributions for the fits of given models to given data sets, which allows the selection of models based on the listed most probable or minimal $\chi^{2}$ values.

### 2.4. Neutron Scattering Experiments

Samples of dry rice pasta with a moisture content of about 0.015 g/g d.m. were chosen for the neutron scattering experiments. The sample material, in the form of a friable powder, was placed into a flat aluminium container. The container was assembled out of two flat (one front and one backside) aluminium plates, each having a cross-section of 64 square centimetres, fully exposed to the incident VESUVIO neutron beam when placed perpendicular to its direction.

The measurements were carried out at −20, 0, 20, 40, 60, and 80 °C, with the temperature stabilized in a closed-cycle refrigerator. The average heating rate was 3.1 °C/min. The general setup of VESUVIO was described elsewhere [21,22,24,30]. The mass-resolved NCS spectra were recorded in the neutron time-of-flight (TOF) domain using the Double Difference technique by detectors placed at scattering angles between 130 and 170 degrees (referred to as the backscattering regime), as well as using the Resonance Difference technique by 48 forward scattering detectors placed at scattering angles between 35 and 70 degrees. The recorded TOF spectra contained the recoil peaks of hydrogen, carbon, and oxygen, as well as the aluminium from the sample container. The traditional protocol of NCS data treatment has been described in great detail elsewhere [20,21,22,23,24,60,61], and thus only the main points will be mentioned here. The NCS spectra were assumed to consist of recoil peaks with underlying purely Gaussian nuclear momentum distributions (NMDs) with standard deviations σ (hereinafter termed as the NMD widths). The set of free fitting parameters consisted of two parameters per atomic species, one NMD width and one relative integral peak intensity. For the NCS data treatment, a multi-step self-consistent iterative algorithm, implemented in the MantidPlot computational environment, was employed [28,40,47,59]. In each step of the data treatment, partitioned non-linear least-squares fits are performed sequentially (on a detector-by-detector basis). Namely, during each iteration of the algorithm for the signal recorded by each detector, for each set of the parameters in which the fitted model is nonlinear (NMD widths), the fitting algorithm solves a linear least-squares problem and finds the optimal values of the relative integral peak intensity values in a unique way using the linear algebra. This feature of the fitting algorithm, together with the fact that peak positions of the fitted NMDs are fixed for each detector signal as a consequence of the conservation of the kinetic energy and momentum in the Compton scattering event, ensures that the fitting results for each detector signal are unambiguous.

For the treatment of neutron transmission data, a bespoke VesuvioTransmission algorithm, implemented in the MantidPlot computational environment, was employed [29,31,32,34]. In the case of high-resolution neutron diffraction data recorded on VESUVIO, the VesuvioDiffractionReduction algorithm, a part of the MantidPlot environment, was used [28,59].

## 3. Results and Discussion

### 3.1. Glass Transition by DSC

**Figure 1:**
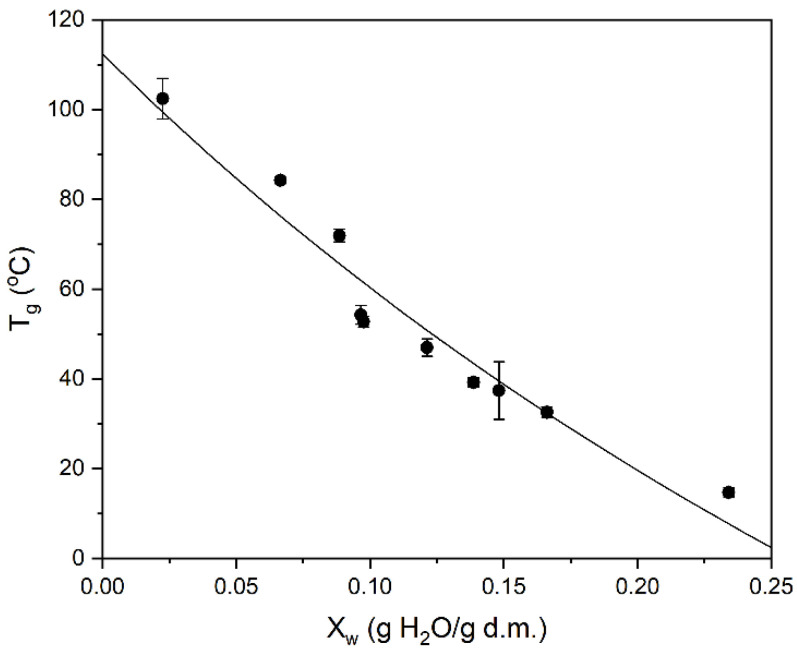
The effect of water content on the $T_{g}$ values measured by DSC. Solid black circles with error bars indicate data points. The solid black line indicates the fit according to the Gordon and Taylor formula given by Equation (3), as described in the text.

The glass transition temperature ($T_{g}$) has been determined as a function of the water content in individual rice pasta samples, and the results are presented in Figure 1. Examples of DSC thermograms are presented in Figure S1 in the Supplementary Materials. For all DSC data under consideration, the $T_{g}$ values decrease with increasing water content. This tendency is related to the plasticizing effect of water, the molecules of which loosen the matrix of pasta. In light of these observations, the experimental data were fitted by the Gordon and Taylor formula:

(3) $$T_{g}=\frac{X_{s}T_{gs}+kX_{w}T_{gw}}{X_{s}+kX_{w}},$$

where $T_{g}$ is the glass transition temperature [°C], $T_{gs}$ is the glass transition temperature of dry matter [°C], $T_{gw}$ is the glass transition temperature of pure water at −135 °C [62], $X_{w}$ is a mass fraction of water, $X_{s}$, a mass fraction of a solid, and k, is the Gordon and Taylor model parameter equal to 2.4. The relatively high value of the coefficient of determination ($R^{2}$), equal to 0.969, indicates that the Gordon and Taylor equation describes the effect of water on lowering the glass transition temperature in the tested product in a very satisfactory manner. The model allows the estimation of the glass transition temperature for the different water content levels of the rice pasta sample.

It is clearly visible in Figure 1 that the range of glass transition temperatures of rice pasta stretches from 105.3 to 13.8 °C, for water content values between 0.0225 and 0.2341 g/g d.m. Notably, the glass transition temperature value, inferred using Equation (3) for the dry rice pasta sample with the water content of 0.1069 g/g d.m., is ca. 55 °C. Moreover, the value of $T_{gs}$ for anhydrous rice pasta, estimated from the Gordon and Taylor model, is 112.4 °C. These values are similar to $T_{g}$ values for rice flour at comparable moisture content, as well as $T_{gs}$ value for the anhydrous sample (117 °C) obtained by Sandoval et al. [63]. On the other hand, the glass transition temperature values determined by Chung et al. [64] for rice starch and for extruded rice pellet, for which $T_{gs}$ values exceeded 150 °C, were higher [65]. Additionally, Perdon et al. [66] and Cao et al. [67] identified three transitions in thermograms for brown rice kernels. Both authors concluded that the increase in water content significantly influenced only the second transition. These findings show that in the investigated rice starch products, the glass transition may be a result of a complicated interplay of the thermal changes and interactions of the starch with proteins and lipids, as well as the origin and method of product preparation.

### 3.2. Glass Transition in Rice Pasta by NMR Analysis

**Figure 2:**
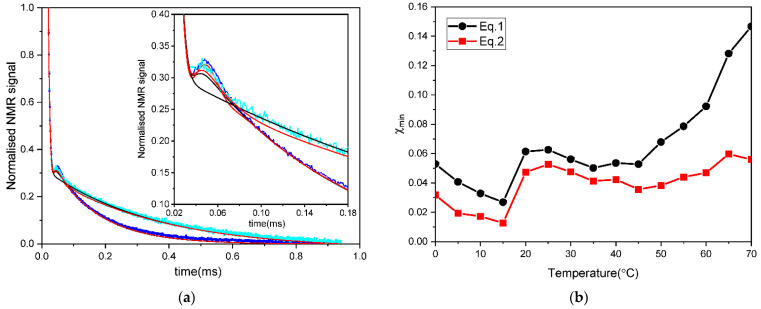
(**a**) The FID signals recorded at 0 °C (blue line and markers) and 70 °C (cyan line and markers) in a rice pasta sample with the water content of 0.1069 g/g d.m. and the fitting curves generated by models described by Equation (1) (solid black line) and Equation (2) (solid red line). (**b**) A plot of $\chi_{min}$ resulting from the fitting of the models described by Equation (1) (solid black points and line) and Equation (2) (solid red squares and line) using FABADA for a set of temperatures between 0 °C and 70 °C (**b**).

Encouraged by the result of the DSC analysis that the glass transition temperature for the dry rice pasta sample with the water content of 0.1069 g/g d.m. is ca. 55 °C, we have chosen the sample with this particular water content to look for signatures of glass transition at the molecular level by acquiring FID signals over a range of temperatures. The signals were fitted according to two models described by Equations (1) and (2) using the FABADA algorithm. The data, together with the fitting curves generated according to both models, are shown in Figure 2a. In Figure 2b, a comparison of the minimal $\chi^{2}$ values $\chi_{min}$, for both models across the entire temperature range, is shown. The model described by Equation (1) has often been used to account for the FID signals in many low-hydrated carbohydrate systems in the glassy state, such as maltose-water [68], glucose syrup with carrageenan [15], starch base mixed with sucrose [17], amylose films with glycerol [16] or films of arabinoxylan and β-glucan [18]. In this two-component model, the rigid (immobile) and flexible (mobile) protons are characterized with second moment $M_{2}$ and transversal relaxation time, $T_{2L}^{*}$ with corresponding amplitudes $S_{s}$ and L, respectively. However, as shown in Figure 2a,b, in rice pasta, this model does not sufficiently fit the line shape of the FID in the whole temperature range. A better fit, as confirmed by smaller $\chi_{min}$ values in the whole temperature range (see Figure 2b), was obtained by Equation (2). In that model, the solid-like proton fraction present in the starch is accounted for by two components, a so-called Abragam function and a Gaussian function. Both functions are meant to account for a fraction of protons exhibiting a degree of local ordering resembling a solid fraction. In such a fraction, strong dipolar interactions between protons are present that do not average out to zero due to the absence of sufficiently fast and isotropic diffusion. Fitting the fast-decaying part of the signal by the two components mentioned above takes into account the complexity of the local solid-like structure, in which amorphous and semi-crystalline starch regions are present, which may interact locally with proteins. The Abragam function, which was originally used to describe FID signals in crystals with strong dipolar couplings [69], is more likely to be associated with semi-crystalline residues in dry pasta. Its contribution to the total signal, according to the value of the parameter denoted as $S_{s}$, is at the level of 0.13 at 25 °C. The Gaussian component typically describes a signal from amorphous disordered phases. The third component of Equation (2) can be attributed to mobile protons, which can be both water and mobile matrix protons, used as exchangeable protons of the polysaccharide [70].

**Figure 3:**
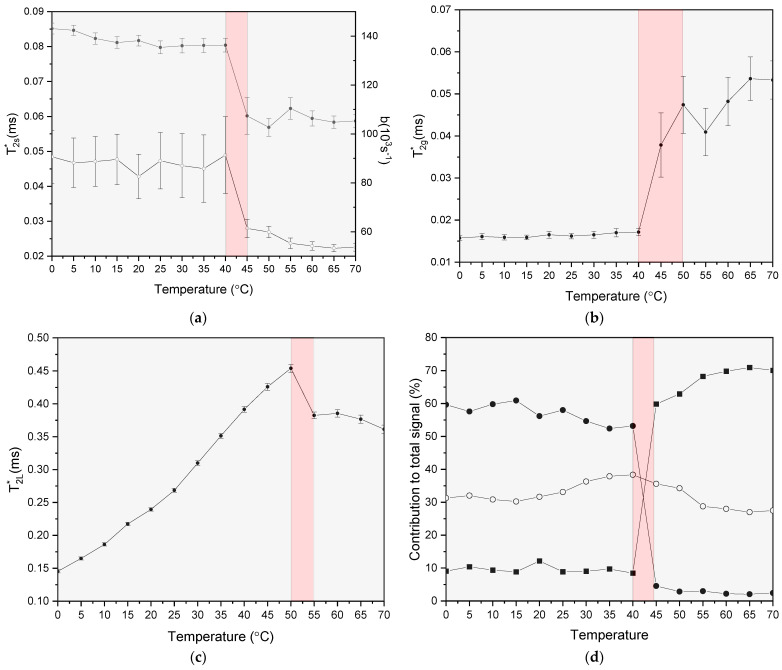
Effects of temperature on NMR relaxation parameters of the model described by Equation (2) for the sample of rice pasta with the water content 0.1069 g/g d.m.: $T_{2s}^{*}$ and b parameter (open and filled circles, respectively) (**a**), $T_{2g}^{*}$ (**b**), $T_{2L}^{*}$ (**c**) and the contribution of individual amplitudes $S_{s}$ (solid black squares), $S_{g}$ (solid black circles), and (open circles) to total solid signal $\left(S_{s}+S_{g}\right)$ (**d**).

As an example, Figure 3a–d shows the effects of temperature on the NMR relaxation parameters of the model described by Equation (2) for a rice pasta sample with the water content of 0.1069 g/g d.m. Significant discontinuities can be noticed in the temperature range 40–45 °C for relaxation times $T_{2s}^{*}$ and $T_{2g}^{*}$, as well as the parameter *b* (Figure 3a,b). Such changes are indicative of a dramatic change in a dynamic state of protons within a solid matrix associated with a complete alteration of their dipolar relaxation parameters. The significant increase of $T_{2g}^{*}$ in this temperature range signifies increased mobility within amorphous regions of rigid starch. Such a change would be expected around the transition between the glass and the rubbery state of the starch, as suggested by NMR state diagrams in many carbohydrate systems [10]. On the other hand, a small drop in values of $T_{2s}^{*}$ at this temperature range evidently reflects the loss of mobility of protons present in residues of semi-crystalline regions. At the same time, a significant decrease in the value of the parameter b in the temperature range 40–45 °C is observed (Figure 2a). Within the Abragam model, such a decrease of the value of b is usually linked to a narrowing of the NMR line shape in the frequency domain, characterizing the degree of the dipolar interaction in the solid-like fraction of protons [71]. The value of the b parameter depends on the number density and distances between protons, and thus its reduction would suggest a decrease of the proton density. As $T_{2s}^{*}$ and b are numerically cross-correlated, one has to treat them together as components of one generalized ‘eigen-parameter’ of the model. Thus, their concurrent changes, as depicted in Figure 3, are indicative of the local-structure changes within the structure of pasta induced by temperature change in the region of 40–45 °C. This picture is also supported by a sharp decrease in the value of the parameter $S_{g}$ and a concomitant increase in the value of $S_{s}$, observed in the same temperature region (Figure 3d). The temperature of the transition for the glassy to the rubbery state in the starch, calculated from DSC measurements using Equation (3), was estimated at 55 °C, a value higher than its counterpart obtained from the analysis of the NMR data. Such a situation is expected, however, due to the complicated nature of the detection of the glass transition by different analytical and spectroscopic methods, especially in the case of heterogeneous food samples [72]. One also cannot exclude that this difference is due to the slightly lower heating rate in NMR experiments compared to DCS measurements, as $T_{g}$ is strongly dependent on the cooling /heating rate. Moreover, a clear trend is observed in the literature, whereby the values of $T_{g}$, obtained from the NMR analysis are several degrees lower than their counterparts obtained for the DSC [13,15,16,18].

Apparently, the value of $T_{g}$ assigned for rice pasta by the DSC analysis better correlates with changes of $T_{2L}^{*}$ values. Namely, as can be seen in Figure 3c, the spin-spin relaxation time for mobile phases constantly increases with temperature and then drops in value for temperatures above 50 °C. The initial increase of the $T_{2L}^{*}$ value is associated with the thermal activation of molecular motions in a fixed local structure. However, above 50 °C, a small but significant decrease indicates that motion of this proton fraction may be a result of cooperative changes within the structures of amorphous and semi-crystalline regions of rice starch. Similar behaviour for spin-spin relaxation time for mobile fraction was observed in previous works [17,18], where its change was attributed to the contribution of motion from polysaccharide protons and/or onset of exchange processes between water protons and protons of hydroxyl groups of the polysaccharides. The acceleration of molecular motion is also related to the increase in the amplitude of the signal from the mobile phase in relation to the constant signal (Figure 3d) up to the temperature range of about 40–45 °C, where the greatest changes in the relaxation parameters of the solid phase occur (Figure 3a–c).

Overall, the results from NMR relaxation experiments indicate significant changes in molecular dynamics induced by structural changes in the dry rice pasta in the temperature range 40–45 °C. Changes in the mobility of protons are observed in both the solid and liquid phase, which is in line with a model in which the polysaccharide structure within the pasta loosens due to the weakening of interactions between protons. The observed mobility changes indicate that the glass transition temperature for the dry rice paste sample under investigation may be in the region of 40–45 °C. The relatively low value of $T_{g}$, inferred from the analysis of the NMR relaxation experiments as compared to the results of the DCS analysis, may be the result of two factors: (i) the slightly lower heating rate in NMR experiments compared to DCS measurements, and (ii) the fact that the total NMR signal may be slightly more weighted towards the signal component coming from protons of water, as the proton spin density is slightly higher in water than in the starch present in the dry rice sample.

### 3.3. Glass Transition by Neutron Experiments

**Figure 4:**
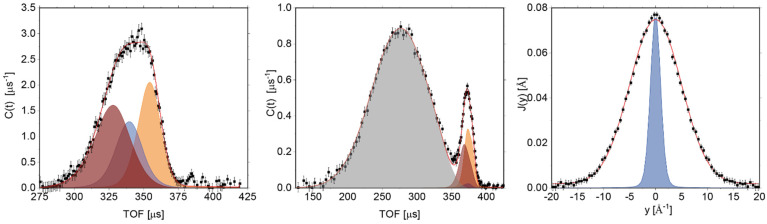
The sums of the atomic mass-resolved TOF NCS spectra and their respective fitted curves representing the underlying Gaussian momentum distributions. The spectra were recorded at 60 °C by the backscattering (**left panel**) and forward scattering (**middle panel**) VESUVIO detectors for the sample of rice pasta with a water content of 0.1069 g/g d.m. The NCS data are shown as solid black points together with error bars. The total fitted functions are shown as solid red lines. The recoil peaks of individual atomic species are shown as shaded areas coloured in wine (for the carbon), blue (for the oxygen), orange (for the aluminium sample container), and grey (for the hydrogen). The (**right panel**) shows the recoil peak of the hydrogen transformed into the hydrogen longitudinal momentum space (full black circles with error bars) and is fitted with a Gaussian momentum distribution (solid red line) together with the instrument resolution function for the protons shown as a blue shaded area.

We start our discussion by showing the results of the analysis of the NCS data of the rice pasta sample with a water content of 0.1069 g/g d.m. As mentioned in the Introduction, NCS is useful in the context of phase changes induced by temperature due to the fact that it provides the relative concentration of each type of atomic species, measured in the units of the total signal. In other words, unlike the NMR technique, which is capable of monitoring the composition of different fractions of the same NMR-active atomic species (e.g., protons in the case of this work), the NCS measures the relative compositions of all types of atomic species present in a given system under investigation simultaneously. Thus, any change of the relative peak intensities observed in an NCS spectrum as a function of temperature would signal a change of sample composition due to a physical change or chemical reaction taking place. To this end, Figure 4 shows the sums of the atomic mass-resolved TOF NCS spectra and their respective fitted curves, representing the underlying Gaussian NMDs, recorded at 60 °C by the backscattering (left panel) and forward scattering (middle panel) detectors. One can clearly see the sums of separate recoil peaks, shown as coloured shaded areas for carbon (wine), oxygen (blue), aluminium from the sample container (orange), and hydrogen (grey). Due to kinematic constraints, the hydrogen peak is only present in NCS data recorded by the forward scattering detectors. Additionally, in the right panel of Figure 4, a proton momentum distribution, J(y), is shown in the domain of the longitudinal proton momentum, y, together with a fit by a Gaussian function of a width almost an order of magnitude larger than the width of the instrument resolution function for the detection of protons, clear evidence of the superb mass resolution of the NCS technique [21,22,23,73,74].

**Figure 5:**
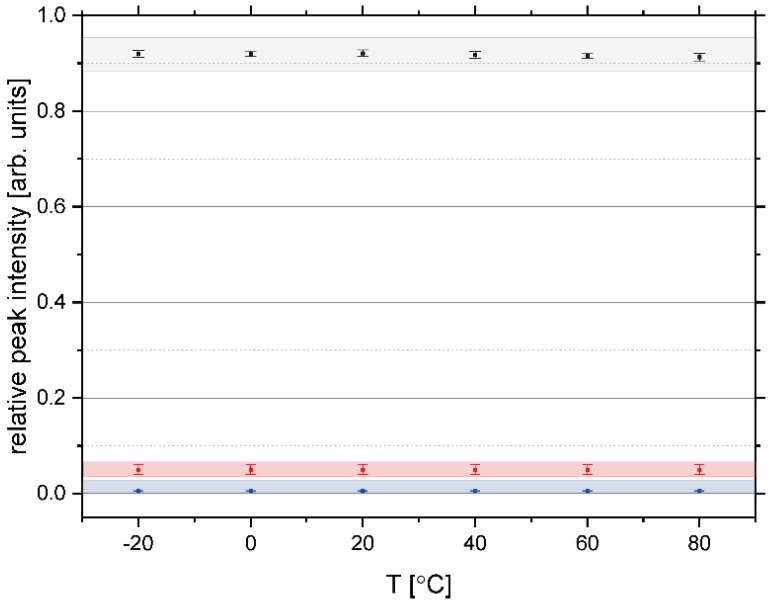
Relative integral intensities of the hydrogen (**black**), carbon (**red**) and oxygen (**blue**), obtained from fitting of the TOF NCS spectra recorded at a set of temperatures in the range of −20 °C to 80 °C for the sample of rice pasta with the water content 0.1069 g/g d.m. The filled points with error bars denote the peak intensities.

Figure 5 shows the relative integral intensities of the hydrogen, carbon, and oxygen, obtained from fitting of the TOF NCS spectra recorded at a set of temperatures in the range of −20 °C to 80 °C for the sample of rice pasta with the water content 0.1069 g/g d.m. As can be clearly seen, the overall stoichiometric composition of the sample does not change within the experimental accuracy across the entire temperature range.

**Figure 6:**
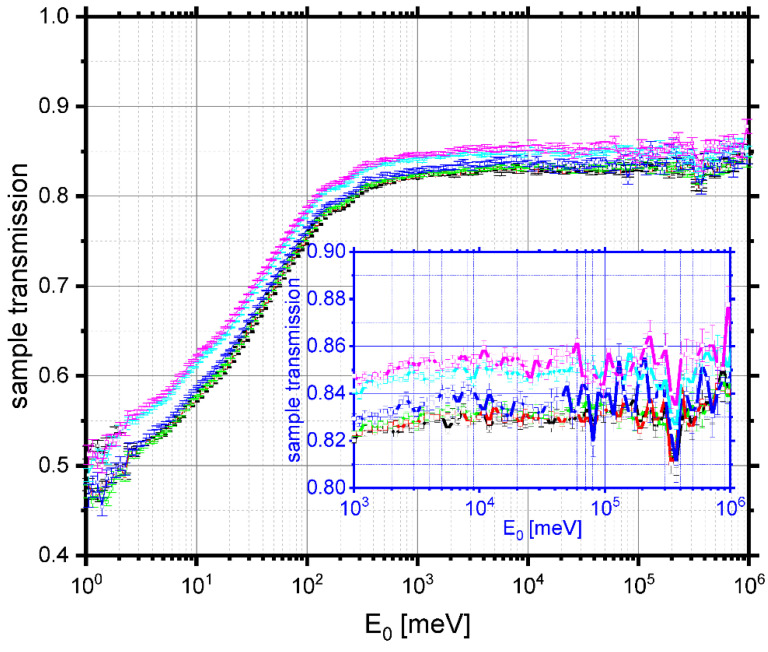
Neutron transmission curves, recorded in a wide range of incident neutron energies, between 1 meV and 1000 eV and at a set of temperatures in the range of −20 °C to 80 °C for the sample of rice pasta with the water content 0.1069 g/g d.m. Black, red, green, blue, cyan, and magenta traces with data points and error bars show data recorded at −20, 0, 20, 40, 60, and 80 °C. Inset: the close-up of the transmission in the epithermal neutron range defining the scattering power of the sample as a function of temperature.

Neutron transmission curves, recorded in a wide range of incident neutron energies, between 1 meV and 1000 eV, are shown in Figure 6. A clear progression is visible of the transmission lines, starting from the bottom (with the lowest magnitude of transmission), for the sample at −20 °C, towards the top (with the largest transmission magnitude), for the sample at 80 °C. Such a progression clearly signifies a systematic increase of transmission of the sample with the increasing temperature.

**Figure 7:**
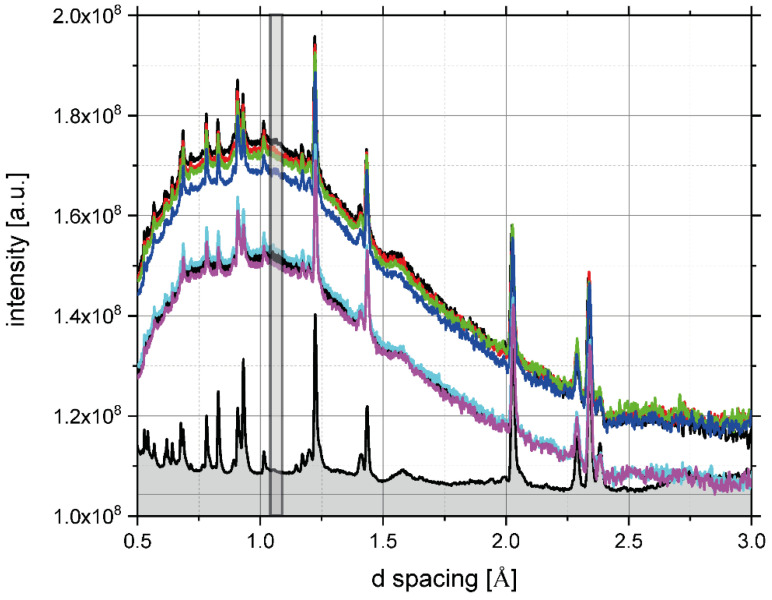
Neutron diffraction curves, recorded for a set of temperatures in the range from −20 °C to 80 °C for the sample of rice pasta with the water content 0.1069 g/g d.m. Black, red, green, blue, cyan, and magenta traces with data points and error bars show data recorded at −20, 0, 20, 40, 60, and 80 °C. The signal from the aluminium sample container is shown as a solid black line with a grey shaded area. The vertical grey rectangle shows the integration area of the incoherent background present in the diffraction spectra of the sample.

Moreover, the clearly observed increase in the magnitudes of the plateau regions of the transmission curves with the increasing temperature signifies a systematic decrease of the sample scattering power with temperature. The scattering power is defined as a product of the sample number density, its thickness, and the total free scattering neutron cross-section weighted by the sample composition [29,31,32,34]. With the sample composition being constant over the entire temperature range under investigation, as evidenced by the NCS data analysis (see Figure 5), the only plausible explanation for such a systematic trend in sample scattering power is that the average sample number density decreases with increasing temperature. In order to corroborate this explanation, in Figure 7, the diffraction curves recorded for the pasta sample placed in aluminium containers are plotted as a function of temperature. The entire incoherent background, present in diffraction curves, is due to the scattering of neutrons on amorphous regions in the sample structure, where no long-range order is present. It is clearly seen in Figure 7 that the only Bragg peaks due to coherent neutron scattering are due to the crystal structure of the aluminium in the sample container. Thus, one can conclude that, in the case of the rice pasta under consideration, the crystallinity is negligible, and the entire detectable sample diffraction signal comes from an amorphous, disordered structure. Our observation is in line with the method proposed to assess the degree of relative crystallinity of starch samples by Nara and Komiya [75,76]. To put this result into perspective, let us mention that, as detailed in Section 2.1, the total carbohydrates in the rice pasta sample were estimated to constitute 86.64 ± 0.01% of its total mass. With the remainder being composed of protein residue, fat, and water, one can safely assume that the only part of the sample that may exhibit some degree of crystallinity is the part attributed to carbohydrates. Moreover, it was established by previous X-ray diffraction studies of different types of starches that the relative crystallinity decreases with decreasing the moisture content of the sample, and for rice starch, at 20% moisture content, the relative crystallinity is at 38% [76]. Additionally, the values of relative crystallinity of various starches that underwent mechanical treatment (ground with a ball mill) were reported to be 0% [76]. Thus, for a rice pasta sample with a water content of 0.1069 g/g d.m., it can be plausibly assumed that the degree of crystallinity is negligible as a result of an interplay of low moisture content and mechanical treatment. It can be clearly seen that the incoherent neutron scattering background level systematically decreases with increasing temperature, a clear signature of systematically decreasing the number density of the amorphous regions of the sample with the increasing temperature.

**Figure 8:**
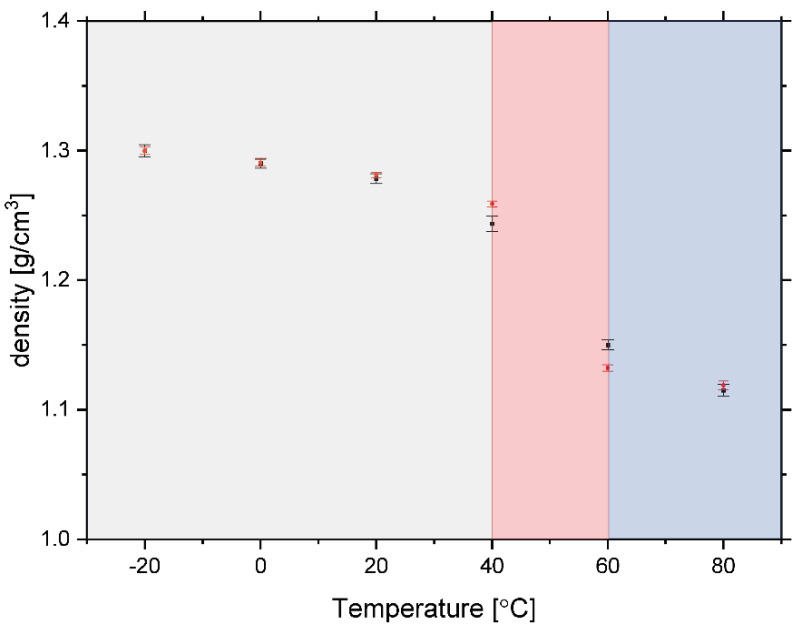
The estimated density values in the range of −20 °C to 80 °C for the sample of rice pasta with the water content 0.1069 g/g d.m., calculated from the data recorded by neutron transmission (solid black points with error bars) and diffraction (solid red points with error bars). See text for details.

Taking both pieces of evidence together, one can plot the anticipated density of the sample as a function of temperature by performing a simple calibration, whereby the level of sample scattering power and the integrated intensity of the incoherent background present in diffraction at the lowest temperature of −20 °C are set to correspond to the tabulated density of starch with a water content of 0.1069 g/g d.m., which is 1.3 g/cm^3^ [77]. The resulting estimated sample density values, calculated from the data recorded by neutron transmission and diffraction, are shown in Figure 8. As can be clearly seen (see the region of Figure 8 marked in red), there is a marked drop in the sample density values, both in the case of data estimated based on neutron transmission and diffraction, in the temperature region between 40 and 60 °C. Interestingly, in the same temperature region, significant changes in molecular dynamics and organization of polysaccharide fraction present in the pasta sample under investigation were observed from the analysis of the NMR relaxation data described above.

Density (or specific volume) change upon change of temperature is one of the most iconic criteria discerning a glassy, amorphous structure from an ordered, crystalline one. Glassy structures are usually formed by rapid (quench) cooling of a liquid, whereas crystalline ones are produced by slow cooling, as a result of which the glasses have lower density [78]. There are known theories describing the behaviour of polymers and other materials near their glass transition temperature range. These theories have been applied to predict the glass transition of foods and pharmaceuticals with some success [78]. One of them is the free-volume theory. The space within the polymer domain in a food sample that is available for rotation and translational movements is considered as a free volume that will favour the mobility of macromolecules. Above the glass transition temperature range, the free volume increases linearly with temperature, as does the mobility of the polymeric molecules [78]. These two observations are completely in line with our findings from the NMR relaxometry, DCS, and neutron experiments performed on the rice pasta sample in this work. Apart from theoretical models, molecular dynamics (MD) has also been recently applied to model the glass transition in systems similar to pasta. The glass transition behaviour of the ternary biopolymeric system consisting of amylose has been modelled by the MD technique by analysing the specific volume as a function of temperature [77]. On the basis of the density MD simulation results, an amorphous cell of 12 monomers with 1.3 g/cm^3^ input density was used in the $T_{g}$ simulation of hydration amylose fragment at different hydration degrees corresponding to the water content between 5.4% and 11.3%. For the hydration level of 11.3%, the drop of the sample density observed in the MD simulations was from a value of ca. 1.3 g/cm^3^ at ca. 42 °C to a value of ca. 1.1 g/cm^3^ at ca. 85 °C (see Figure 6d in Ref. [77]). Those figures compare very favourably with the values reported in our study in Figure 8. Thus, it is plausible to assume that all three types of techniques employed in this study witness a glass transition occurring in the amorphous part of the pasta sample in the temperature range between 40 and 60 °C.

**Figure 9:**
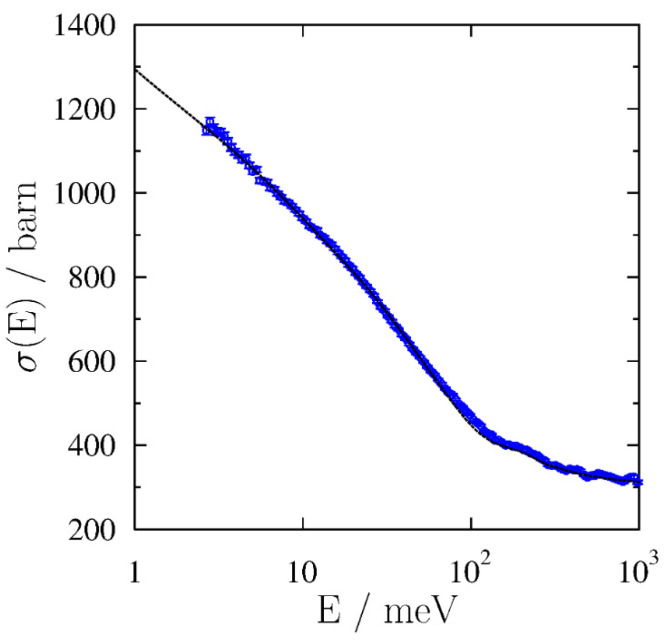
The total neutron scattering curve, recorded as a function of the incident neutron energy for the sample of rice pasta with the water content 0.1069 g/g d.m. at 40 °C together with the prediction from the AFGA model. The experimental data are shown as blue markers and the AFGA model prediction as a dashed black line.

In order to shed more light on the vibrational structure changes accompanying the proposed glass transition in rice pasta starch between 40 and 60 °C, we have treated the transmission data using a recently developed methodology [37]. Namely, it has been clearly shown that the total neutron scattering curves of many organic materials, recorded in the incident neutron range between 1 meV and 1000 eV, can be rationalised in terms of the average contributions of different functional groups, thus neglecting their correlation, an approach termed as Average Functional Group Approximation (AFGA) [37]. The AFGA method is well suited for the particular case of the low-water content highly mechanically treated rice pasta, where the crystallinity level, coming from branched amylopectin polymers, is negligible, and the entire structure is dominated by an amorphous, glassy state. To this end, the transmission data, recorded for the rice pasta with the water content 0.1069 g/g d.m., have been analysed and interpreted assuming that the stoichiometry of the sample corresponds to a mixture of a polymer of α-glucose units, (C_6_H_10_O_5_)_n_ and water, C_6_H_10_O_5_ (H_2_O)_1.1_. The use of the AFGA approximation has allowed the construction of synthetic total neutron scattering curves for the same set of temperatures at which the real rice pasta sample transmission curves were recorded. Each synthetic curve was built under the assumption that functional groups present in the polymer of α-glucose units, i.e., the CH_2_, OH and CH, have distinctive, independent contributions, and their linear combination forms the total cross-section curve of the α-glucose units. Similarly, it was assumed that the total neutron scattering curve of water can be decomposed into the contributions of one oxygen atom and two hydrogen atoms in the OH group. Importantly, due to the incoherent nature of the AGFA approximation, one can safely assume that the same dissection scheme into functional group contributions to the total neutron scattering curves of the rice pasta starch under consideration can be applied to describe both the linear α-glucose polymer, the amylose, forming the amorphous matrix within starch, and to the branched α-glucose polymer, the amylopectin, forming the crystalline domains within starch. However, residual in size and number these crystalline domains could be in a highly dehydrated and mechanically treated sample. Moreover, the approximation of the sample with the C_6_H_10_O_5_ (H_2_O)_1.1_ stoichiometry has allowed correcting the neutron transmission data for the varying density of the sample and expressing the results as an effective cross-section measured in units of barns. A result of this procedure for the total neutron cross-section curve recorded at 40 °C is shown in Figure 9.

As can be clearly seen in Figure 9, despite a relatively coarse approximation of the entire pasta sample, treating it as a mixture of a polymer of α-glucose units water and neglecting the other organic residue (proteins, fatty acids), the prediction of the AFGA model is extremely good, a testimony to the fact that the dominant contributions to the total neutron cross-section stem from the incoherent inelastic scattering off hydrogen, and that the latter is well modelled by the considered functional groups.

**Figure 10:**
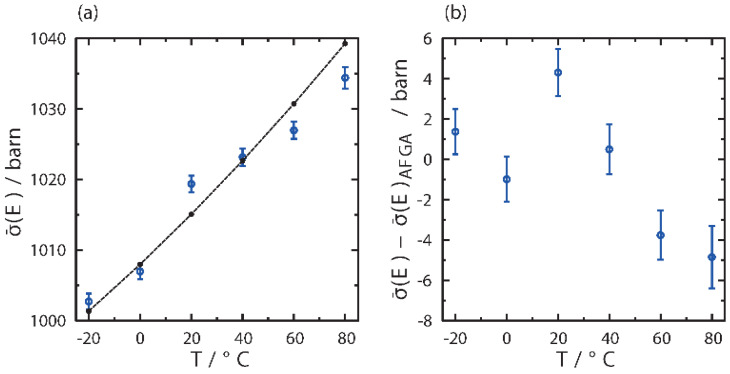
(**a**) The integrals, in the incident neutron energy range between 2.6 and 10 meV, of the total neutron scattering curves, for the sample of rice pasta with the water content 0.1069 g/g d.m. between −20 and 80 °C together with the integrals of the predictions from the AFGA model. The integrals of the experimental data are shown as blue markers and the integrals of the AFGA model predictions as a dashed black line. (**b**) The difference between the integrals of the experimental total neutron scattering cross-sections and their counterparts resulting from the predictions of the AFGA model.

In order to get additional insight into the vibrational structure of the rice pasta sample under investigation, one has to take into account that neutron transmission data always exhibit much more marked dependence on the atom-projected vibrational densities of states (apVDOSes) in the low incident neutron energy region [26,29,31,32,34,35,36]. To this end, the neutron transmission data have been integrated into the incident neutron energy window, in which the temperature changes are most apparent, i.e., between 2.6 and 10 meV. The results of this procedure are shown in Figure 10a.

Clearly, Figure 10a shows that the AFGA model almost exactly follows the experimental data up to ca. 40 °C, and then starts to underestimate the data, with the amount of discrepancy being of the order of 0.5% in the temperature region between 60 and 80 °C. In order to visualise this discrepancy even better, Figure 10b shows the difference between experiment and AFGA prediction, thus factoring out the temperature effect. It is clearly seen in Figure 10b that the difference between the experimental data and the prediction of the AFGA model forms a similar trend as a function of temperature as the changes in the sample density visible in Figure 8, as well as the changes in NMR relaxation parameters depicted in Figure 3. In order to explain this systematic discrepancy, let us start with the remark that, within the AFGA model, a purely incoherent sum of individual functional group contributions is taken, without any consideration for any elements of the secondary structure. In the case of the rice pasta, the AFGA model does not take into account any changes in the residual amount of branching within the amylopectin domains in the starch, as well as structural changes in the amorphous part of the starch, the amylose, occurring with temperature, such as loosening of the network structure of entangled linear α-glucose units, increasing the void spaces between them, and the overall transition from a glassy to a rubbery state. Instead, the AGFA model takes into account solely the temperature-driven changes in the populations of vibrational modes within apVDOSes of individual atomic species present in the functional groups such as CH_2_, OH and CH. Thus, the differences between the AFGA model prediction and the experimental values, clearly visible above 40 °C, are most likely due to changes in the secondary structure within the amorphous regions in rice starch that are accompanied by the breaking of chemical bonds. With some bonds broken, the vibrational structure of the starch changes and the apVDOSes of individual atomic species present in the functional groups under consideration exhibit depleted intensity regions corresponding to the low-energy modes missing. In consequence, the total neutron cross-section values decrease below the level predicted by the AFGA model. Importantly, changes in the vibrational structure of starch due to changes in the secondary structure and relative crystallinity level have been reported in the literature [79,80,81,82,83,84,85]. Specifically, for the low-energy modes, terahertz time-domain spectroscopy and low-frequency Raman scattering were performed on the natural polymer starch to investigate the boson peak (BP) dynamics. In the infrared spectrum, the BP was observed at 0.99 THz (ca. 4.13 meV) at the lowest temperature, with both the frequency of the BP and absorption coefficient showing lower values than those of the vitreous glucose [85]. These secondary structure-induced changes in the low-energy part of the apVDOSes were explained to originate from the longer correlation length of the medium-range order and lower concentration of hydroxyl groups in the starch, compared to the vitreous glucose [85]. Overall, it is thus quite plausible to assume that the changes in the low-energy vibrational structure of the rice pasta starch are dictated by the changes in the secondary structure, whereby, as the glass transition progresses, the glassy structure disintegrates under the influence of temperature and is progressively replaced by more rubbery structure.

**Figure 11:**
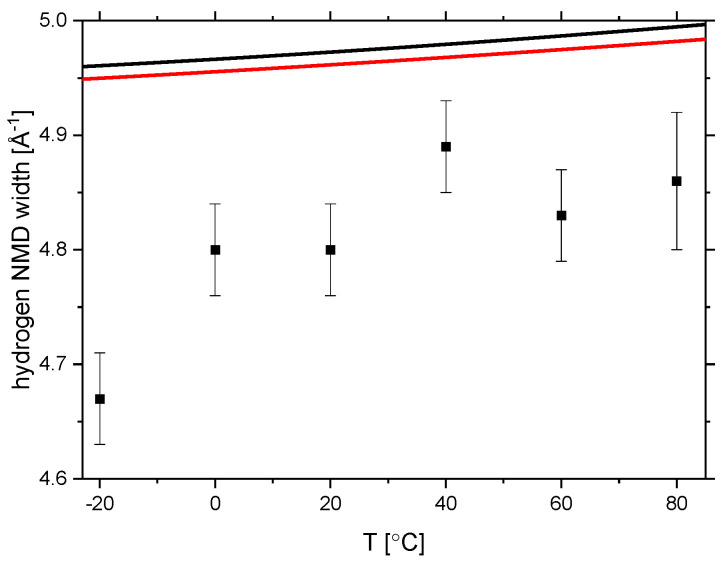
The NMD widths of hydrogen obtained from NCS experiments for the sample of rice pasta with the water content 0.1069 g/g d.m. between −20 and 80 °C. The thick black line represents the NMD width computed based on the AFGA model for a polymer of α-glucose units, (C_6_H_10_O_5_)_n_. The thick red line represents the NMD width computed based on the AFGA model for a mixture of a polymer of α-glucose units, (C_6_H_10_O_5_)_n_ and water, C_6_H_10_O_5_ (H_2_O)_1.1_.

In order to complete the assessment of the temperature-induced changes in the vibrational structure of the rice pasta starch under investigation, in Figure 11, we contrast the values of NMD width of the hydrogen obtained from the NCS experiments with their counterparts calculated from two AFGA models, one assuming that the sample consists only of the polymer of α-glucose units, (C_6_H_10_O_5_)_n_, and the other assuming a mixture of (C_6_H_10_O_5_)_n_ and water, corresponding to the water content 0.1069 g/g d.m., C_6_H_10_O_5_ (H_2_O)_1.1_. As can be clearly seen, despite the fact that the AFGA approximation for a model containing water molecules describes the experimental data slightly better, both AFGA models yield almost constant values of NMD widths of the protons due to the Boltzmann population factor being to a good approximation equal to unity in this temperature range.

Importantly, the discrepancy between the experimental data and the AFGA predictions clearly decreases with increasing temperature. According to the theory of NCS, the values of NMD widths are most influenced by the changes in the high-energy parts of apVDOSes of the respective nuclei [21,22]. Thus, the convergence of the AFGA model towards the experimental NMD values is most likely driven by the changes of the high-energy regions in the apVDOS of the hydrogen that intensify with increasing temperature. Those changes are due to the increased share in the apVDOS of the high-energy stretching and bending modes pertaining to molecular vibrations of isolated or fragmented molecular moieties that the AFGA model very well describes. Interestingly, such changes have been reported in the literature. For example, various starch samples varying in molecular structure, organization, and moisture content were studied by ATR-FTIR spectroscopy [80]. The comparison of the infrared spectra showed that changes in band intensities in the 1065–870 cm^−1^ region could be explained by the mobility changes in the starch related to the glass transition. Taken together, the systematic shift of the centre-of-gravity of the apVDOS of the hydrogen in the starch sample and the increased ability of the AFGA model to account for the values of the proton NMD widths, both observed with increasing temperature, can only be reconciled with a model in which the secondary structure of the starch breaks down and increased fragmentation of the polymer structure occurs during the glass transition.

On the whole, the picture of the vibrational structure changes dictated by the changes in the secondary structure of the rice pasta starch, painted by the concurrent application of neutron diffraction, transmission, and Compton scattering, is a complicated one. Upon the glass transition between 40 and 60 °C, both low and high-energy parts of apVDOSes of atomic species within the relevant functional groups change due to bond breaking and reorganisation of glucose polymers present in the amylose and amylopectin in starch.

## 4. Conclusions

In the first part of our study, the glass transition has been characterized at a macroscopic level by a number of techniques. To this end, the results obtained by neutron diffraction and DSC revealed that dry rice pasta is almost entirely amorphous. Moreover, the glass transition was evidenced by neutron transmission and diffraction data and manifested as a significant decrease of the average sample number density in the temperature range between 40 and 60 °C. Importantly, the NCS data analysis revealed no changes in the average sample composition in the entire temperature range under consideration, thus providing an additional degree of assurance that the changes in the levels of signals observed in neutron diffraction and transmission pertain solely to density changes across temperature. Finally, the observed density change across the glass transition was in qualitative and quantitative agreement with the results of molecular dynamics studies reported in the literature, linking changes in local starch structure across the glass transition to a marked decrease in the average sample density and providing an important link between the macroscopic behaviour and the structure and dynamics at the microscopic level.

At the microscopic level, our study has provided evidence of changes in the secondary structure of the starch within the dry rice pasta. These changes have been detected by the NMR relaxometry as increased mobility of protons in the amorphous regions of the sample and concurrent changes in dynamic parameters describing residues of semi-crystalline regions. Moreover, the changes in the values of the spin-spin relaxation times recorded by NMR across the glass transition correlated very well with the systematic decrease in the sample number density observed by neutron transmission and diffraction. The changes of the secondary starch structure, observed by NMR, have also been evidenced by a careful analysis of the neutron transmission data by means of the average functional group approximation method. Interestingly, the same method has proven to be useful in detecting the changes in the secondary starch structure across the glass transition as observed by NCS.

Overall, this work has extended the usual arsenal of the experimental techniques used to study glass transition by employing, apart from NMR relaxometry and DSC, the neutron scattering-based techniques, neutron transmission, diffraction, and Compton scattering. This particular combination of techniques has enabled the characterisation of the structural and dynamical signatures of glass transition in dry rice pasta in an unprecedented range of spatio-temporal scales, alleviating some methodological problems usually encountered in studies of the glass transition by means of traditional techniques and rendering the obtained results less model-dependent. Moreover, the concurrent application of neutron transmission, diffraction, and Compton scattering on the same neutron beamline, VESUVIO thermal-to-epithermal neutron station, has minimized the influence of systematic sources of errors on the data analysis.

## Data Availability

The data presented in this study are available upon reasonable request from the corresponding author. The raw neutron data collected at ISIS Neutron and Muon Source are related to the RB number 1990362.

## Supplementary Materials

The following are available online at https://www.mdpi.com/article/10.3390/polym13152426/s1, Figure S1: DSC thermograms showing glass transition of rice pasta samples of different moisture content.
