# Supplementary material for: Glass Transition in Rice Pasta as Observed by Combined Neutron Scattering and Time-Domain NMR
Source: Polymers (Basel). 2021 Jul 23;13(15):2426. doi: 10.3390/polym13152426 (PMC8347043; doi:10.3390/polym13152426)
Supplement: Supplementary file 1 [file polymers-13-02426-s001.zip › polymers-1288164-supplementary.pdf]

polymers

MDPI

Supplementary Material

Glass Transition in Rice Pasta as Observed by Combined Neutron Scattering and Time-Domain NMR

Magdalena Witek [1], Maciej Krzystyniak [2,*], Giovanni Romanelli[2] and Teresa Witczak [3]

[1] Department of Biotechnology and General Technology of Food, Faculty of Food Technology, University of Agriculture in Krakow, Balicka 122, 30-149 Krakow, Poland
[2] ISIS Facility, Rutherford Appleton Laboratory, Chilton, Didcot, OX11 0QX, United Kingdom
[3] Department of Engineering and Machinery for Food Industry, Faculty of Food Technology, University of Agriculture in Krakow, Balicka 122, 30-149 Krakow, Poland
* Correspondence matthew.krzystyniak@stfc.ac.uk; Tel.: +44 78-25-307-959

Content

Figure S1. DSC thermograms showing glass transition of rice pasta samples of different moisture content.

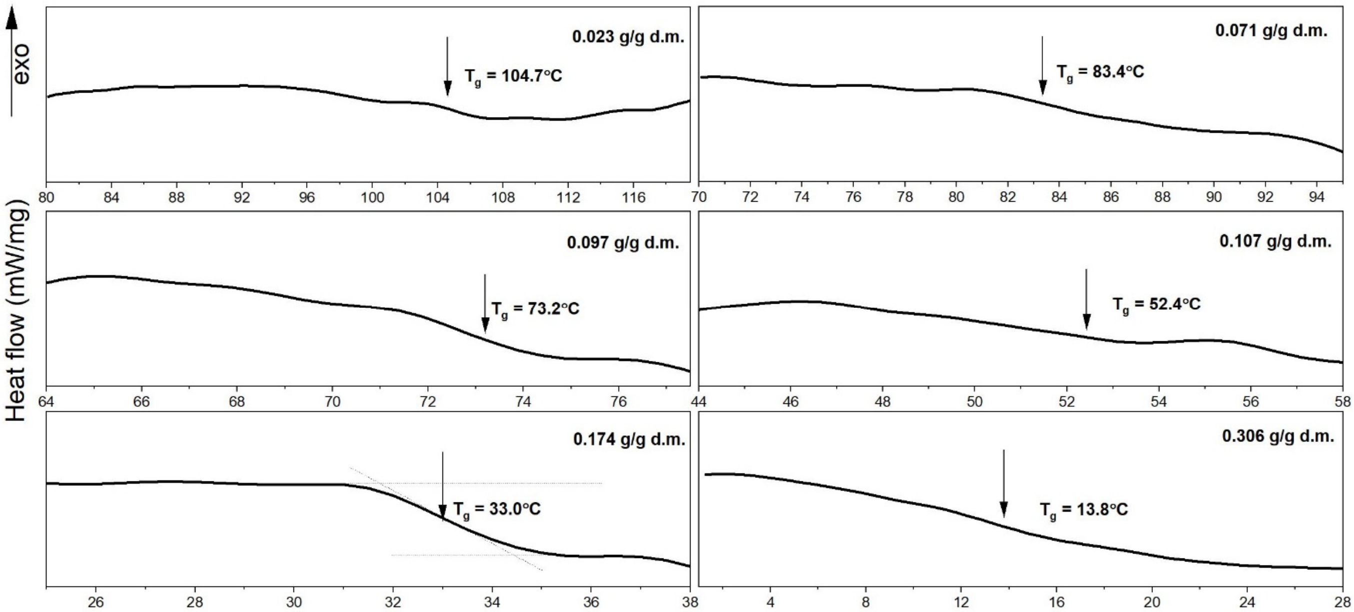

Figure S1. DSC thermograms showing glass transition of rice pasta samples of different moisture content.
